# Adipose Tissue Insulin Resistance Is Positively Associated With Serum Uric Acid Levels and Hyperuricemia in Northern Chinese Adults

**DOI:** 10.3389/fendo.2022.835154

**Published:** 2022-06-10

**Authors:** Honglin Sun, Xiaona Chang, Nannan Bian, Yu An, Jia Liu, Song Leng, Guang Wang

**Affiliations:** ^1^ Department of Endocrinology, Beijing Chao-yang Hospital, Capital Medical University, Beijing, China; ^2^ Health Management Center, The Second Hospital of Dalian Medical University, Dalian, China

**Keywords:** adipose tissue insulin resistance, Adipo-IR, HOMA-IR, uric acid, hyperuricemia

## Abstract

**Objective:**

Adipose tissue plays a crucial role in serum uric acid (UA) metabolism, but the relative contribution of adipose tissue insulin resistance (IR) to serum UA levels and hyperuricemia have not explicitly been illustrated. Herein, we aimed to investigate the association between the adipose tissue insulin resistance index (Adipo-IR) and hyperuricemia in this cross-sectional study. The homeostasis model assessment of insulin resistance (HOMA-IR) index, another widely applied marker to determine systemic IR, was also explored.

**Methods:**

A total of 5821 adults were included in this study. The relationship between Adipo-IR or HOMA-IR and serum UA levels was assessed by multivariate linear regression. Binary logistic regression analyses were applied to determine the sex-specific association of the Adipo-IR tertiles and HOMA-IR tertiles with hyperuricemia. Participants were then divided into normal BMI (18.5 ≤ BMI < 24) and elevated BMI (BMI ≥ 24) groups for further analysis.

**Results:**

Both Adipo-IR and HOMA-IR were positively correlated with serum UA (*P* < 0.001). Compared with the lowest tertile, the risks of hyperuricemia increased across Adipo-IR tertiles (middle tertile: OR 1.52, 95%CI 1.24-1.88; highest tertile: OR 2.10, 95%CI 1.67–2.63) in men after full adjustment (*P* for trend < 0.001). In women, only the highest tertile (OR 2.09, 95%CI 1.52-2.87) was significantly associated with hyperuricemia. Those associations remained significant in participants with normal BMI status. As for HOMA-IR, only the highest tertile showed positive relationships with hyperuricemia in both genders after full adjustment (*P* for trend < 0.001). The association between HOMA-IR and hyperuricemia disappeared in men with normal BMI status.

**Conclusions:**

Adipo-IR was strongly associated with serum UA and hyperuricemia regardless of BMI classification. In men with normal BMI, Adipo-IR, rather than HOMA-IR, was closely associated with hyperuricemia. Altogether, our finding highlights a critical role of adipose tissue IR on serum UA metabolism and hyperuricemia.

## Introduction

Uric acid (UA), the final degradation metabolite of endogenous and exogenous purine, is produced from the liver, intestine, and muscles and mainly excreted through the kidney (1, 2). Hyperuricemia, a serious threat to public health, is attributable to the imbalance between UA synthesis and clearance (3). Hyperuricemia is not only the precursor of gout and kidney stones but also the obvious risk factor of metabolic syndrome, hypertension, cardiovascular disease, and chronic kidney disease (1, 3–5). Recent studies confirmed that adipose tissue was another major organ responsible for UA production (2, 6); meanwhile, the causal role of adiposity in hyperuricemia has been established in many studies, highlighting the importance of adipose tissue on UA metabolism (7, 8). Regardless, there are no existing studies specifically addressing the exact role of insulin resistance (IR) in adipose tissue played in serum UA levels and hyperuricemia.

Adipose tissue is an insulin-sensitive organ essential for glucose and lipid metabolism (9). Under normal circumstances, insulin stimulates glucose uptake and lipogenesis while suppressing the lipolysis of fat cells. Adipose tissue IR is marked with the blunted antilipolytic effect of insulin and consequently increased FFA release from adipose tissue (10). Excessive circulation FFA delivery to liver and muscles thereby results in ectopic fat storage in these organs and subsequent hepatic and muscle IR. Therefore, the assessment of adipose tissue IR is vital as it is the early metabolic defect prior to systematic IR. The determination of lipolysis fluxes by isotope-labeled tracing through the multistep pancreatic clamp and hyperinsulinemic–euglycemic clamp techniques used to be the gold-standard measurement methods for accessing adipose tissue insulin sensitivity, which is somehow expensive, time consuming, and inapplicable to large-scale populations (11, 12). Recently, a surrogate index, adipose insulin resistance (Adipo-IR) index, calculated as fasting plasma free fatty acid (FFA) (mmol/L) × fasting plasma insulin (FINS) mIU/L concentrations, has been validated to be a unique, simple and reliable predictor of adipose tissue IR in obesity-related metabolic disorders (11–15). For instance, our previous study confirmed that Adipo-IR progressively increased from overweight to class III obesity (15). In addition, Adipo-IR has been proven to be the major determent of hepatic fat content and the fibrosis degree of Nonalcoholic fatty liver disease (NAFLD) (10, 16). Adipo-IR is also correlated with abnormal glucose intolerance and metabolic syndrome (9, 13). Results from the San Antonio Metabolism Study and two other prospective studies revealed that Adipo-IR rose progressively during the development of Type 2 diabetes mellitus (17–19). A prospective study further indicated that elevated Adipo-IR was associated with a higher risk of incident dysglycemia (20). Nonetheless, the association between Adipo-IR and hyperuricemia has never been elucidated.

Different from Adipo-IR, the homeostatic model assessment of insulin resistance (HOMA-IR), calculated as fasting plasma glucose [(FBG) (mmol/L) × FINS mIU/L)]/22.5, is a widely applied parameter that focuses on glucose metabolism and mainly introduced to demonstrate systemic IR, especially the hepatic IR (21–23). Although a high correlation between the two indexes has been observed, recent studies indicated their discordance in reflecting whole-body metabolism (24, 25). Compared to HOMA-IR, Adipo-IR was more related to visceral obesity, serum TG, and liver fibrosis (24). A previous study has indicated the association of HOMA-IR with hyperuricemia (21, 26). Nevertheless, the distinct association of the two indexes with serum UA levels and hyperuricemia has not been compared and elucidated.

This study aimed to demonstrate the impact of adipocyte IR on serum UA and hyperuricemia by using the Adipo-IR index for the first time, additionally, to compare the distinct roles of Adipo-IR and HOMA-IR played in hyperuricemia in both male and female subjects with a different BMI status.

## Methods

### Study Population

This cross-sectional study included 5,925 participants in Northern China who underwent routine physical examination between April 2017 and August 2021 in Beijing Chao-yang Hospital. We excluded participants under 18 years old (n = 5), with missing UA data (n = 8), missing HbA1c value (n = 10), missing blood pressure value (n = 66), and with reduced renal dysfunction (n = 15), a final number of 5,821 subjects were included in the final analysis. Ethical approval was obtained from the ethics committee of Beijing Chao-yang Hospital. All participants signed written informed consent in the study.

### Anthropometric and Biochemical Measurements

Height, body weight, systolic blood pressure (SBP), and diastolic blood pressure (DBP) were measured by the same trained team using standard methods as previously described (27). The venous blood samples were collected after overnight fasting. Serum UA levels were determined using a Siemens Advia 2400 biochemical analyzer (Siemens Healthcare Diagnostics Inc., Tarrytown, New York, USA). The FFA, total cholesterol (TC), triglyceride (TG), low-density lipoprotein cholesterol (LDL-C), high-density lipoprotein cholesterol (HDL-C), and serum creatinine (Scr) were measured by colorimetric enzymatic assays using a biochemical auto-analyzer (Hitachi 7170). FBG, FINS, and glycated hemoglobin (HbA1c) levels were detected as previously described (28). The estimated glomerular filtration rate (eGFR) was calculated as previously described (29).

### Definition of Variables

Hyperuricemia was defined as serum UA ≥ 420 μmol/L in men and ≥ 360 μmol/L in women. Hypertension was identified as SBP ≥ 140 mmHg, DBP ≥ 90 mmHg, or a self-reported previous diagnosis of hypertension by a physician. Diabetes was determined as FBG ≥ 7.0 mmol/L, HbA1c ≥ 6.5% or self-reported previous diagnosis of diabetes by a physician. Dyslipidemia was defined as TC ≥ 6.22 mmol/L, TG ≥ 2.26 mmol/L, LDL-C ≥ 4.14 mmol/L, HDL-C < 1.04 mmol/L, or a self-reported previous diagnosis of hyperlipidemia by a physician. Adipo-IR was calculated by the formula: Adipo-IR= FFA (mmol/L) × FINS (mIU/L). HOMA-IR was calculated by the following formula: HOMA-IR = FBG (mmol/L) × FINS (mIU/L)/22.5. Body mass index (BMI) was calculated as weight divided by height squared (kg/m^2^). Participants were divided into normal weight (18.5 ≤ BMI < 24) and elevated weight (BMI ≥ 24) subgroups as previously described (30).

### Statistical Analysis

The IBM SPSS Statistics software, version 25 (IBM Corporation, Armonk, NY, United States), the Graphpad 7.0 software and the MedCalc version 17 software were used for data analysis. Basic characteristic analysis was conducted in participants with hyperuricemia and non-hyperuricemia. We performed analysis separately in men and women to avoid potential sex influences on the proportion of hyperuricemia. The Shapiro–Wilk test was used for normality test and data were expressed as mean ± standard deviation (SD) for continuous normally distributed variables, median (upper and lower quartiles) for continuous skewed distributed variables and number (%) for categorical variables in this study. The difference of normally distributed variables between two groups was calculated with unpaired Student’s *t* test. The Mann–Whitney U test and Kruskal–Wallis test were applied to compare the difference of continuous skewed variables between two groups or three groups, separately. For categorical data, the chi-square test was used as appropriate for categorical variables. The linear trends of hyperuricemia proportion across the Adipo-IR and HOMA-IR tertiles were tested by the Cochran Armitage trend test.

Sex-specific linear regression analysis was accessed to explore the association of serum UA (dependent variable) with Adipo-IR and HOMA-IR (independent variable). UA was Ln-transformed for analysis due to skewed distribution. The variables without collinearity were selected for adjustment. Model 1 was without adjustment; Model 2 was adjusted for age, BMI, SBP, TC, H-DLC, TG, HbA1c, and eGFR; and Model 3 was conducted by excluding participants with diabetes and was adjusted for Model 2.

Adipo-IR and HOMA-IR were then divided into tertiles, Adipo-IR, male: lowest tertile ≤ 2.87; middle tertile 2.88–5.30; highest tertile ≥ 5.30; Female: lowest tertile ≤ 2.96; middle tertile 2.97–5.11; highest tertile ≥ 5.11; HOMA-IR, male: lowest tertile ≤ 1.44; middle tertile 1.45–2.41; highest tertile ≥ 2.41; Female: lowest tertile ≤ 1.25; middle tertile 1.26–2.01; highest tertile ≥ 2.01. The sex-specific associations of Adipo-IR tertiles or HOMA-IR tertiles with the prevalence of hyperuricemia were detected by binary logistic regression analyses, with the lowest tertile as the reference. Model 1 was crude, model 2 was adjusted for age, BMI, HbA1c, eGFR, hypertension and dyslipidemia. Model 3 was conducted by excluding participants with diabetes and was adjusted for Model 2. Further analyses were conducted in BMI subgroups (normal BMI and elevated BMI). Age, HbA1c, eGFR, hypertension, and dyslipidemia were adjusted. Additionally, the association of per SD increase of both indexes with hyperuricemia were analyzed, respectively. Data were summarized as odds ratios (ORs) and 95% confidence intervals (CIs). Furthermore, we use the receiver operating characteristic (ROC) curve analysis to compare the predictive powers of the two indexes for hyperuricemia among men and women. The comparison of the area under the curve (AUC) was analyzed by Delong’s ROC test. For the above analysis, Two-tailed *P* values <0.05 were considered statistically significant.

## Results

### Characteristics of Participants With Hyperuricemia and Non-Hyperuricemia

As presented in **Table 1**, the prevalence of hyperuricemia was 35.2% in men and 13.3% in women. The Adipo-IR and HOMA-IR and BMI levels were all higher in participants with hyperuricemia than individuals without hyperuricemia in both genders (*P* < 0.001). Female subjects with hyperuricemia were older and were more prone to have metabolic disorders such as diabetes, hypertension, and dyslipidemia than non-hyperuricemia subjects (all *P* < 0.001). Interestingly, male participants with hyperuricemia were significantly younger (*P* < 0.001) and the proportion of individuals with diabetes was smaller (*P* = 0.012) compared to non-hyperuricemia subjects. Additionally, the proportions of individuals with hypertension were comparable among male participants with and without hyperuricemia (*P* = 0.232).

**Table 1:** Basic characteristics of the participants with and without hyperuricemia.

Variable	men			Women		
	Normal	Hyperuricemia	*P*-value	Normal	Hyperuricemia	*P-*value
*N* (%)	1,950 (64.8)	1,060 (35.2)		2,436 (86.7)	375 (13.3)	
Age, years	46.76 ± 12.85	42.96 ± 12.56	< 0.001	43.39 ± 13.29	47.64 ± 15.22	<0.001
BMI, kg/m^2^	25.16 ± 3.17	26.73 ± 3.45	< 0.001	22.76 ± 3.45	25.30 ± 4.50	<0.001
DBP, mmHg	126.04 ± 16.50	128.11 ± 16.79	0.001	117.54 ± 18.24	125.01 ± 21.07	<0.001
SBP, mmHg	75.49 ± 11.37	77.33 ± 12.02	< 0.001	68.63 ± 11.03	72.06 ± 11.64	<0.001
FBG, mmol/L	4.96 (4.60-5.44)	4.97 (4.58-5.43)	0.850	4.76 (4.43-5.12)	4.98 (4.59-5.45)	<0.001
HbA1c, %	5.5 (5.3-5.8)	5.5 (5.3-5.8)	0.505	5.4 (5.2-5.7)	5.6 (5.3-6.0)	<0.001
FINS, mIU/L	7.7 (5.4-10.7)	9.7 (6.7-13.7)	< 0.001	7.2 (5.3-9.9)	10.0 (6.5-14.3)	<0.001
FFA, mmol/L	0.46 (0.35-0.60)	0.50 (0.39-0.62)	< 0.001	0.52 (0.40-0.67)	0.60 (0.46-0.75)	<0.001
TG, mmol/L	1.36 (0.98-1.92)	1.74 (1.22-2.50)	< 0.001	1.01 (0.77-1.39)	1.41 (0.96-2.12)	<0.001
TC, mmol/L	4.89 (4.31-5.51)	5.09 (4.53-5.72)	< 0.001	4.86 (4.31-5.52)	5.23 (4.59-5.99)	<0.001
H-DLC, mmol/L	1.18 (1.00-1.35)	1.10 (1.00-1.30)	< 0.001	1.50 (1.27-1.71)	1.30 (1.10-1.50)	<0.001
L-DLC, mmol/L	3.00 (2.48-3.60)	3.20 (2.67-3.73)	< 0.001	2.80 (2.30-3.36)	3.14 (2.55-3.89)	<0.001
UA, μmol/L	357.0 (320.0-388.0)	468.0 (441.0-508.0)	< 0.001	273.0 (240.0-307.0)	390,0 (373.0-418.8)	<0.001
eGFR, mL/min per 1.73 m^2^	111.32 (102.82-120.07)	111.26 (100.89-120.41)	0.290	116.57 (106.62-126.18)	107.97 (99.56-121.51)	<0.001
Adipo-IR	3.50 (2.23-5.52)	4.87 (3.04-7.31)	< 0.001	3.73 (2.44-5.62)	5.67 (3.48-9.18)	<0.001
HOMA-IR	1.74 (1.17-2.53)	2.19 (1.44-3.21)	< 0.001	1.53 (1.08-2.16)	2.27 (1.39-3.39)	<0.001
Hypertension, *n* (%)	430 (22.1)	254 (24.0)	0.232	316 (13.0)	90 (24.0)	<0.001
Diabetes, *n* (%)	229 (11.7)	93 (8.8)	0.012	96 (3.9)	30 (8.0)	<0.001
Dyslipidemia, *n* (%)	861 (44.2)	626 (59.1)	< 0.001	524 (21.5)	167 (44.5)	<0.001

Data were expressed as the mean ± SD or median (upper and lower quartiles) or number (proportion). BMI, body mass index; SBP, systolic blood pressure; DBP, diastolic blood pressure; FPG, fasting plasma glucose; HbA1c, glycated hemoglobin; FINS, fasting insulin level; FFA, free fatty acid; TG, triglycerides; TC, total cholesterol; HDL-C, high-density lipoprotein cholesterol; LDL-C, low-density lipoprotein cholesterol; UA, uric acid; eGFR, estimated glomerular filtration rate; Adipo-IR, adipose tissue insulin resistance; HOMA-IR, homeostasis model assessment of insulin resistance.

### Association of Serum UA Levels With Adipo-IR or HOMA-IR Index by Linear Regression Analysis

**Figure 1:**
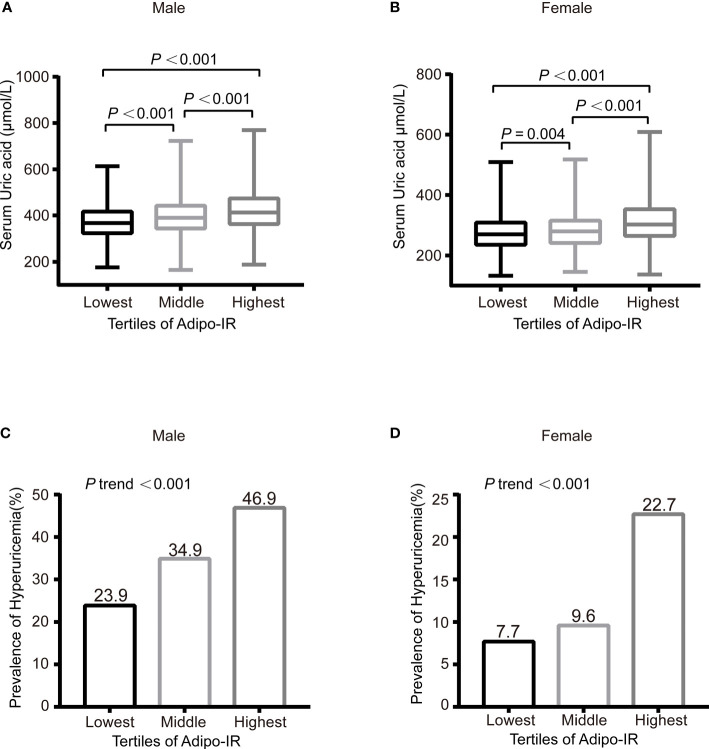
The serum UA levels **(A**, **B)** and prevalence of hyperuricemia **(C**, **D)** across the Adipo-IR tertiles. Data were expressed as median (upper and lower quartiles) or proportion (%). *P* trend: from test for linearity.

The correlation between Adipo-IR or HOMA-IR and serum uric acid by spearman analysis was presented in **Supplementary Table 1**. Both Adipo-IR and HOMA-IR showed positive correlations with UA in both genders (all *P* < 0.001). The correlations existed in both normal BMI and elevated BMI subgroups (all *P* < 0.001). Further linear regression analysis indicated that higher Adipo-IR or HOMA-IR levels were associated with higher Ln UA levels in both genders (**Table 2**) (all *P* < 0.001). The positive correlations remained in both genders after full adjustment (all *P* < 0.001). As the proportion of diabetes was distinct in men and women, we excluded participants with diabetes and found consistent positive relationships between both the indexes and Ln UA in both genders (all *P* < 0.001). Furthermore, the serum UA levels (all *P* < 0.01) as well as the ratio of hyperuricemia (*P* for trend < 0.001) exhibited increasing trends from the lowest to highest tertiles of the two indexes in both genders (**Figure 1** and **Supplementary Figure 1**).

**Table 2:** Linear regression analysis for association of Adipo-IR and HOMA-IR (independent variables) with LnUA (dependent variable).

Variable	Ln UA (men)				Ln UA (women)	
	*B*(SE)	Standardized β	*P-*value	*B*(SE)	Standardized β	*P-*value
**Adipo-IR**						
Model 1	0.013 (0.001)	0.235	<0.001	0.015 (0.001)	0.266	<0.001
Model 2	0.007 (0.001)	0.125	<0.001	0.009 (0.001)	0.151	<0.001
Model 3	0.008 (0.001)	0.127	<0.001	0.009 (0.001)	0.146	<0.001
**HOMA-IR**						
Model 1	0.016 (0.002)	0.145	<0.001	0.033 (0.003)	0.235	<0.001
Model 2	0.007 (0.002)	0.064	0.001	0.016 (0.003)	0.114	<0.001
Model 3	0.009 (0.003)	0.059	0.004	0.022 (0.004)	0.117	<0.001

Model 1: Crude model.

Model 2: Adjusted for age, BMI, SBP, TC, H-DLC, TG, HbA1c, and eGFR.

Model 3: Excluding participant with diabetes and adjusted for Model 2.

### Association of Adipo-IR and HOMA-IR With Hyperuricemia by Logistic Regression Analyses

The association of sex-specific Adipo-IR and HOMA-IR tertiles with the prevalence of hyperuricemia is shown in **Table 3**. The lowest tertile was used as the reference. Overall, the risk of hyperuricemia increased across Adipo-IR tertiles and HOMA-IR tertiles (all *P* for the trend <0.001). In the crude model, the ORs of middle and highest Adipo-IR tertiles for hyperuricemia in men were 1.71 (95% CI 1.41–2.08) and 2.82 (95% CI 2.33–3.41), respectively. After adjusting for confounders, the ORs of the middle and highest Adipo-IR tertiles for hyperuricemia were 1.52 (95% CI 1.24–1.88) and 2.10 (95% CI 1.67–2.63), respectively. Among women, only the OR of the highest Adipo-IR tertile for hyperuricemia was statistically significant compared to the lowest tertile, either in a crude (OR 3.53, 95%CI 2.66–4.70) or fully adjusted model (OR 2.09, 95%CI 1.52–2.87) (all *P* for trend < 0.001). Similar relationships between Adipo-IR and hyperuricemia were observed in non-diabetic participants. Furthermore, we observed a 24% and 47% higher risk of hyperuricemia with each SD increment in Adipo-IR in men and women, respectively.

**Table 3:** Logistic regression analysis for association between Adipo-IR or HOMA-IR and hyperuricemia.

Variables	men			Women		
	Model 1	Model 2	Model 3	Model 1	Model 2	Model 3
**Adipo-IR**						
Lowest tertile	Ref.	Ref.	Ref.	Ref.	Ref.	Ref.
Middle tertile	1.71(1.41,2.08)***	1.52(1.24,1.88)***	1.49(1.20,1.85)***	1.28(0.92,1.77)	1.04(0.74,1.46)	1.02(0.72,1.44)
Highest tertile	2.82(2.33,3.41)***	2.10(1.67,2.63)***	2.07(1.64,2.62)***	3.53(2.66,4.70)***	2.09(1.52,2.87)***	2.02(1.46,2.79)***
* P* for trend	<0.001	<0.001	<0.001	<0.001	<0.001	<0.001
Per SD increase	1.45(1.34,1.57)***	1.24(1.13,1.36)***	1.33(1.19,1.49)***	1.75(1.59,1.93)***	1.47(1.31,1.64)***	1.52(1.34,1.72)***
**HOMA-IR**						
Lowest tertile	Ref.	Ref.	Ref.	Ref.	Ref.	Ref.
Middle tertile	1.41(1.17,1.71)***	1.14(0.92,1.40)	1.13(0.91,1.39)	1.11(0.81,1.53)	0.92(0.66,1.29)	0.85(0.61,1.20)
Highest tertile	2.32(1.93,2.80)***	1.61(1.28,2.03)***	1.63(1.28,2.07)***	3.20(2.42,4.23)***	1.74(1.26,2.42)**	1.60(1.15,2.24)**
* P* for trend	< 0.001	< 0.001	< 0.001	< 0.001	< 0.001	< 0.001
Per SD increase	1.25(1.16,1.35)***	1.07(0.98,1.17)	1.19(1.04,1.37)*	1.72(1.54,1.93)***	1.43(1.25,1.64)***	1.58(1.32,1.89)***

Model 1: Crude model.

Model 2: Adjusted for age, BMI, HbA1c, eGFR, hypertension, and dyslipidemia.

Model 3: Excluding participant with diabetes and adjusted for Model 2.

*P < 0.05; **P < 0.01; ***P < 0.001.

The meaning of the bold values is the values were statistically significant.

As for the HOMA-IR index, similar to Adipo-IR, the highest tertile of HOMA-IR showed higher hyperuricemia risk in women (Model 1: OR 3.20, 95%CI 2.42–4.23; Model 2: OR 1.74, 95%CI 1.26–2.42; Model 3: OR 1.60, 95%CI 1.15–2.24). Meanwhile, in men, different from Adipo-IR, only the highest tertile HOMA-IR (OR 1.61, 95%CI 1.28–2.03) showed a positive relationship with hyperuricemia after full adjustment.

### Association of Adipo-IR Tertiles and HOMA-Tertiles With Hyperuricemia by Logistic Regression Analyses in Normal BMI and Elevated BMI Subgroups

**Figure 2:**
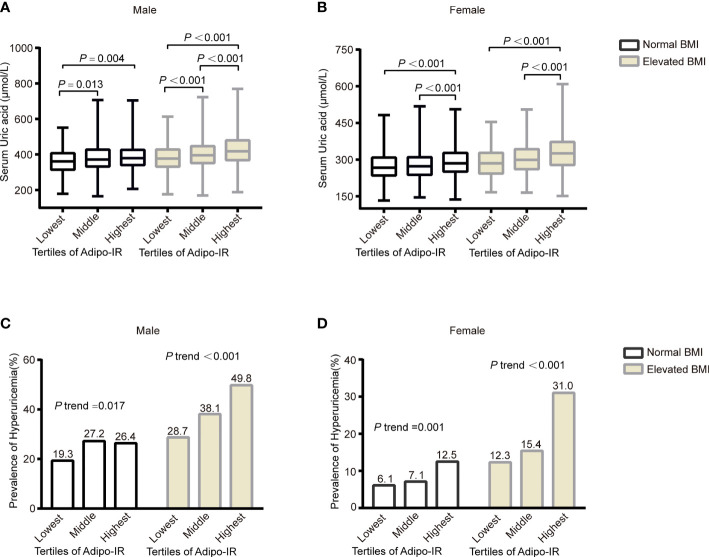
The serum UA levels **(A, B)** and prevalence of hyperuricemia **(C**, **D)** across the Adipo-IR tertiles in normal BMI and elevated BMI subgroups. Data were expressed as median (upper and lower quartiles) or proportion (%). *P* trend: from test for linearity.

**Figure 3:**
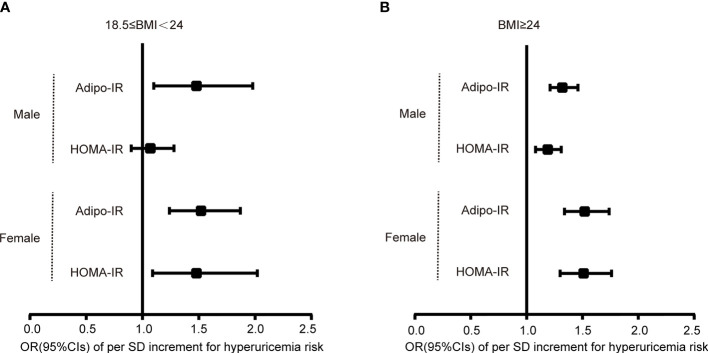
Logistic regression analysis for the OR of per SD increment of Adipo-IR or HOMA-IR for hyperuricemia risk in normal BMI **(A)** and elevated BMI **(B)** subgroups. Models were adjusted for age, HbA1c, eGFR, hypertension, and dyslipidemia. .

To evaluate the association of Adipo-IR and HOMA-IR with hyperuricemia among participants with normal weight, we conducted a subgroup analysis in participants with normal BMI or elevated BMI status. The UA levels and the proportion of subjects with hyperuricemia showed an increasing trend from the bottom to top Adipo-IR and HOMA-IR tertiles even in normal BMI subgroups of both genders (**Figure 2** and **Supplementary Figures 2–4**) (*P* for trend < 0.001). In the normal BMI subgroup, one SD increase in Adipo-IR showed 48% (*P* for trend = 0.025) and 52% (*P* for trend = 0.002) higher risks for hyperuricemia in men and women, respectively. By contrast, one SD increase in HOMA-IR was not associated with higher hyperuricemia risk in men (**Figure 3**) (**Table 4**). When Adipo-IR and HOMA-IR were entered as tertiles, both the middle and highest Adipo-IR tertiles showed significantly positive association with hyperuricemia in men irrespective of BMI classification. In contrast, no association was observed between HOMA-IR tertiles and hyperuricemia in male normal BMI subgroup (**Table 4**).

**Table 4:** Logistic regression analysis for association between Adipo-IR or HOMA-IR and hyperuricemia in both normal BMI and elevated BMI subgroups.

Variables	men		Women	
	18.5 ≤ BMI < 24	BMI ≥ 24	18.5 ≤ BMI < 24	BMI ≥ 24
**Adipo-IR**				
Lowest tertile	Ref.	Ref.	Ref.	Ref.
Middle tertile	1.63(1.13,2.35)**	1.59(1.23,2.06)***	1.12(0.70,1.78)	1.23(0.70,2.15)
Highest tertile	1.65(1.01,2.70)*	2.63(2.03,3.40)***	1.99(1.25,3.17)**	2.81(1.70,4.63)***
* P* for trend	0.025	< 0.001	0.002	< 0.001
Per SD increase	1.48(1.10,1.98)**	1.32(1.21,1.46)***	1.52(1.24,1.87)***	1.52(1.34,1.74)***
**HOMA-IR**				
Lowest tertile	Ref.	Ref.	Ref.	Ref.
Middle tertile	1.39(0.97,2.01)	1.11(0.86,1.44)	0.96(0.61,1.51)	1.00(0.56,1.81)
Highest tertile	1.58(0.91,2.76)	1.85(1.42,2.40)***	1.88(1.18,3.00)**	2.03(1.20,3.45)**
* P* for trend	0.020	<0.001	0.002	<0.001
Per SD increase	1.07(0.90,1.28)	1.19(1.08,1.31)**	1.48(1.09,2.02)*	1.51(1.30,1.76)*

Models were adjusted for age, HbA1c, eGFR, hypertension and dyslipidemia.

*P < 0.05; **P < 0.01; ***P < 0.001.

The meaning of the bold values is the values were statistically significant.

### The AUC of Adipo-IR and HOMA-IR for Hyperuricemia

**Supplementary Figure 5** displays the ROC curves related to the diagnostic ability of Adipo-IR and HOMA-IR for hyperuricemia incidence among men and women, respectively. In women, the AUC for Adipo-IR (0.667, 95%CI 0.649–0.684) and HOMA-IR (0.654, 95%CI 0.636–0.672) were comparable (*P* = 0.18), whereas in men, Adipo-IR showed larger AUC (0.624, 95%CI 0.607–0.642) than HOMA-IR (0.604, 95%CI 0.586–0.622) (*P* = 0.003), which implied a closer relationship between Adipo-IR and hyperuricemia.

## Discussion

This is the first study exploring the relationship between Adipo-IR and UA as well as hyperuricemia in a Northern Chinese population. Our study revealed that Adipo-IR was positively associated with serum UA levels and hyperuricemia. This relationship was independent of age, BMI, eGFR, diabetes, dyslipidemia, and hypertension. Furthermore, we found that HOMA-IR was not as good as Adipo-IR to predict hyperuricemia in men, especially in a normal BMI status, indicating a possibly closer relationship between Adipo-IR and serum UA metabolism compared to hepatic insulin resistance.

Adipose tissue is an essential endocrine organ for UA metabolism. Theoretically, xanthine oxidoreductase (XOR) is the responsible enzyme for the final step of UA production. Apart from the small intestine and liver, adipose tissue is another major organ with abundant expression and activities of XOR and is indispensable for UA production and secretion (6, 31). The causal role of obesity to elevated serum UA levels has been well established for years (7, 8). Genetically, adiposity was also considered to be positively associated with serum UA concentrations and the risk of gout (7, 8). Consistent with this, XOR was reported to be a regulator of adipogenesis and correlated positively with adipose mass. Obese adipose tissues own higher XOR activities and thereby possess a higher ability for UA secretion (31). The elevated XOR activity during obesity may be related to hypoxia and active lipid metabolism consuming nicotinamide adenine dinucleotide phosphate. Reciprocally, long-term elevated UA levels could result in a pro-inflammatory state of adipose tissue by inducing monocyte chemotactic protein 1 release and thus causing a vicious cycle (32).

IR has been suggested to be the mediator between obesity and hyperuricemia (33). The amelioration of IR decreased the serum UA level independent of weight loss, while the UA-lowering therapy did not change insulin sensitivity in hyperuricemia subjects (34, 35). By contrast, other studies indicated that hyperuricemia could be detected before hyperinsulinemia (3, 36).In a prospective study, elevated serum UA could predict IR in 15 follow-up years (4). Although current studies indicated a reciprocal causation between IR and hyperuricemia, it is doubtless that compensatory hyperinsulinemia is the bridge between IR and hyperuricemia (37). Compensated hyperinsulinemia caused by IR could decrease the renal clearance of UA, as has long been proven (26, 38, 39). Admittedly, both compensated hyperinsulinemia during hepatic IR or adipose IR could contribute to reduced UA excretion. It is noteworthy that adipose IR precedes hepatic IR or systemic IR during the course of obesity. Current evidence has revealed that visceral obesity, more closely linked to adipose dysfunction, was more responsible for hyperuricemia than overall adiposity (40, 41). Although subcutaneous fat constitutes a major proportion of total fat, visceral fat contributes more to serum UA levels. The possible explanation may be that visceral fat has a stronger lipolysis ability and attributes to adipose IR to a greater degree. Firstly, compensatory hyperinsulinemia caused by excess visceral fat IR could decrease the renal clearance of UA as described above. Secondly, a stronger lipolysis of visceral adipose tissue can increase the flow of FFA to the liver, accelerating the *de novo* lipogenesis in the liver. The increased need for nicotinamide adenine dinucleotide phosphate (NADPH) in this process is accompanied by activated PPP pathways and purine synthesis. Therefore, the above process resulted in the acceleration of hepatic UA production. Consistently, studies indicated that Adipo-IR increased proportionally with visceral fat (41). In our study, we firstly used the simple Adipo-IR index to demonstrate the role of IR in adipose tissue played in UA metabolism and found a positive relationship between them beyond total adiposity, further implying the pathogenic effect of adipose tissue dysfunction in serum UA homeostasis.

As a simple serum index, Adipo-IR has been validated against the cumbersome isotope- tracing experiment for accurately accessing adipose insulin sensitivity. Adipo-IR was shown to be positively correlated with BMI (15, 18, 25). Additionally, previous studies indicated that the Adipo-IR index could reflect the state of adipose tissue IR only in subjects with overweight and obesity (11, 14). Recently, growing studies indicated that Adipo-IR could mirror metabolism disorders regardless of obesity. Adipo-IR showed strong correlations with both the hepatic fat and fibrosis of NAFLD patients independently of BMI and Type 2 diabetes mellitus (10, 42). Obese subjects with normal insulin-sensitive adipose tissue seldom develop ectopic fat deposition in the liver (43). In our study, Adipo-IR was shown to be an independent risk factor for hyperuricemia even in participants with normal BMI, indicating the critical role of adipose tissue function beyond fat mass on UA metabolism.

Theoretically and practically, Adipo-IR is highly correlated with HOMA-IR (25, 32, 42). However, the discordance between the two indexes for demonstrating metabolic diseases has gained great attention recently (24, 44). One study claimed that elevated Adipo-IR was more closely linked with visceral adiposity and hypertriglyceridemia, while elevated HOMA-IR was associated with a lower basic metabolic rate (24). Another study indicated that Adipo-IR was more closely associated with developing prediabetes relative to HOMA-IR (13, 18), indicating a more initial role of adipose tissue IR for glucose dysregulation. Furthermore, compared to HOMA-IR, Adipo-IR was more related to the severity of fibrosis in NAFLD and could be more critical for aortic valve calcification (10, 45). These inconsistencies are possibly due to the differential metabolic effects of adipose tissue and other metabolic organs, more importantly, the pathogenic role of adipose tissues in those diseases.

Several studies indicated a positive relationship between HOMA-IR and hyperuricemia (21). It was not equivalent to the exact role of adipose tissue IR on hyperuricemia. In addition, the present study revealed that Adipo-IR was more closely related with hyperuricemia than HOMA-IR, verifying a more essential role of adipose tissue IR on serum UA metabolism, at least in men. One explanation may be that FFA outweighs glucose for the initiation of hyperuricemia. Another factor that cannot be ignored is sex hormones. Consistent with our results, L.-K. Chen et al. claimed a positive association between serum UA and HOMA-IR in older women but not in men among a Taiwan population (46). Another study revealed a sex-difference association between metabolically healthy obese status and hyperuricemia (47). Further studies are warranted to explore the sex-related roles of IR in hyperuricemia.

Previous studies indicated a bell-shaped relationship between HbA1c, FBG, and serum UA levels (1, 2, 48, 49). UA was positively correlated with FBG and HbA1c before the onset of diabetes (2). However, in individuals with diabetes, UA would be decreased as urine glucose facilitated the excretion of UA (49). Therefore, we also evaluated the relationship between Adipo-IR and uric acid in the non-diabetes participants. We got the conclusion that Adipo-IR correlated well with serum UA levels irrespective of the blood glucose status.

There are some limitations to this study. First of all, the direct causal relationship between Adipo-IR and hyperuricemia cannot be inferred from the observational association in this research. Secondly, the serum UA level is also affected by other confounding factors, such as alcohol consumption, purine-rich diets, diuretic therapy, and genetic risk (4, 7, 50), which we did not collect in this study. Furthermore, the variables that reflect the content of visceral fat, such as waist circumference or the percentage of the body fat, were not collected in this study. Finally, all the participants were of yellow race. This may limit the generalizability of our results to other races. Consequently, more studies are needed to demonstrate the causality, and to extend our findings in different ethnicities and regions.

## Conclusion

To summarize, this is the first study to explore the association of Adipo-IR and serum UA as well as hyperuricemia. Our study indicated a critical role of adipose tissue IR on serum UA metabolism in both normal weight and overweight/obesity participants.

## Data Availability Statement

The raw data supporting the conclusions of this article will not be made publicly available because the ethical approval obtained for this study prevents the human data being shared publicly to protect patients’ privacy. Requests to access the datasets should be directed to GW, wangguang@bjcyh.com.

## Ethics Statement

The studies involving human participants were reviewed and approved by the Ethics Committee of Beijing Chao-yang Hospital affiliated with Capital Medical University. The patients/participants provided their written informed consent to participate in this study.

## Author Contributions

GW and SL designed the study. HS, XC, NB, YA, and JL conducted the research. HS, XC, and NB analyzed the data. HS and XC wrote the manuscript. The final manuscript was read and approved by all authors.

## Funding

This study was not supported by any sources of funding.

## Conflict of Interest

The authors declare that the research was conducted in the absence of any commercial or financial relationships that could be construed as a potential conflict of interest.

## Publisher’s Note

All claims expressed in this article are solely those of the authors and do not necessarily represent those of their affiliated organizations, or those of the publisher, the editors and the reviewers. Any product that may be evaluated in this article, or claim that may be made by its manufacturer, is not guaranteed or endorsed by the publisher.
