# Supplementary material for: Adipose Tissue Insulin Resistance Is Positively Associated With Serum Uric Acid Levels and Hyperuricemia in Northern Chinese Adults
Source: Front Endocrinol (Lausanne). 2022 Jun 10;13:835154. doi: 10.3389/fendo.2022.835154 (PMC9226335; doi:10.3389/fendo.2022.835154)
Supplement: Supplementary file 1 [file DataSheet_1.docx]

Supplementary Material

# Supplementary Tables

**Supplementary Table 1. Correlation between serum UA and Adipo-IR or HOMA-IR using Spearman correlation analysis**

|  | **Serum UA (Male)** | | **Serum UA (Female)** | |
| --- | --- | --- | --- | --- |
| **Variables** | **r** | ***P*** | **r** | ***P*** |
| **All** |  | | | |
| Adipo-IR | 0.262 | < 0.001 | 0.245 | < 0.001 |
| HOMA-IR | 0.214 | < 0.001 | 0.238 | < 0.001 |
| **18.5 ≤ BMI < 24** |  | | | |
| Adipo-IR | 0.169 | < 0.001 | 0.115 | < 0.001 |
| HOMA-IR | 0.154 | < 0.001 | 0.092 | < 0.001 |
| **BMI ≥ 24** |  | | | |
| Adipo-IR | 0.231 | < 0.001 | 0.287 | < 0.001 |
| HOMA-IR | 0.148 | < 0.001 | 0.245 | < 0.001 |

UA, uric acid; Adipo-IR, adipose tissue insulin resistance Index; HOMA-IR, homeostasis model assessment of insulin resistance.

# Supplementary Figures


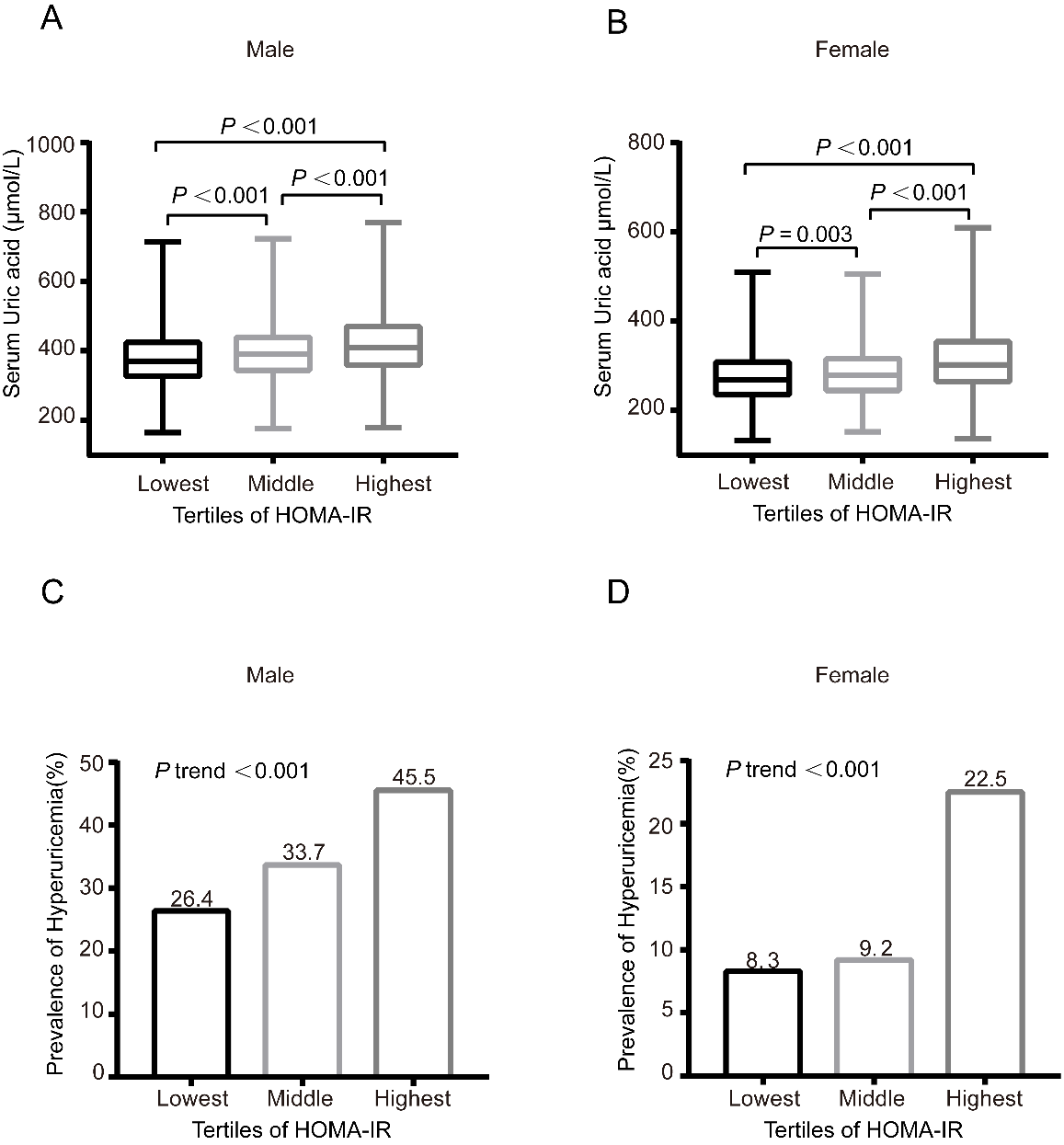


**Supplementary Figure 1. The serum UA levels (A and B) and prevalence of hyperuricemia (C and D) across the HOMA-IR tertiles.** Data were expressed as median (upper and lower quartiles) or proportion (%). *P* trend: from test for linearity.


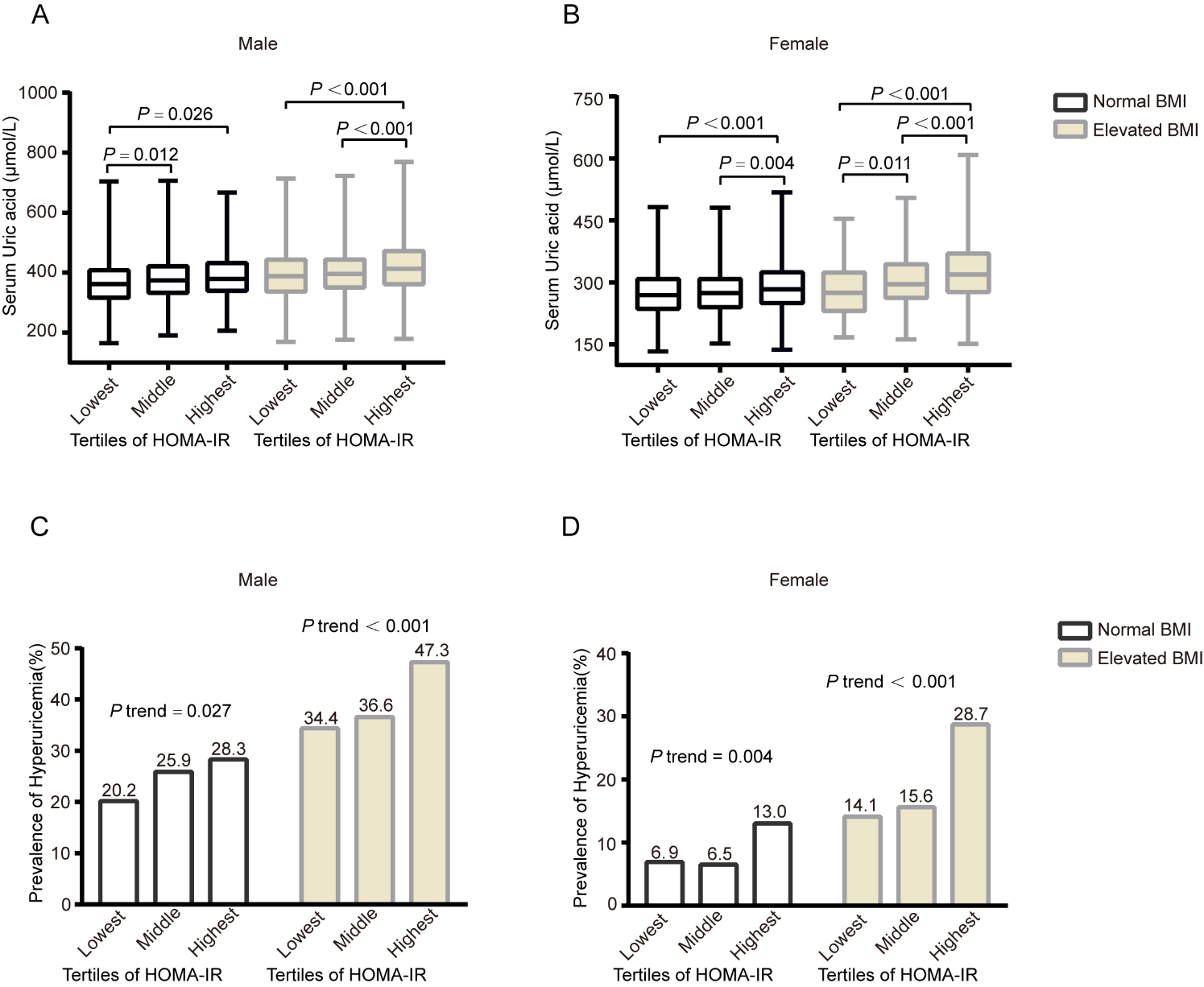


**Supplementary Figure 2. The serum UA levels (A and B) and prevalence of hyperuricemia(C and D) across the HOMA-IR tertiles in normal BMI and elevated BMI subgroups.** Data were expressed as median (upper and lower quartiles) or proportion (%). *P* trend: from test for linearity.

~~
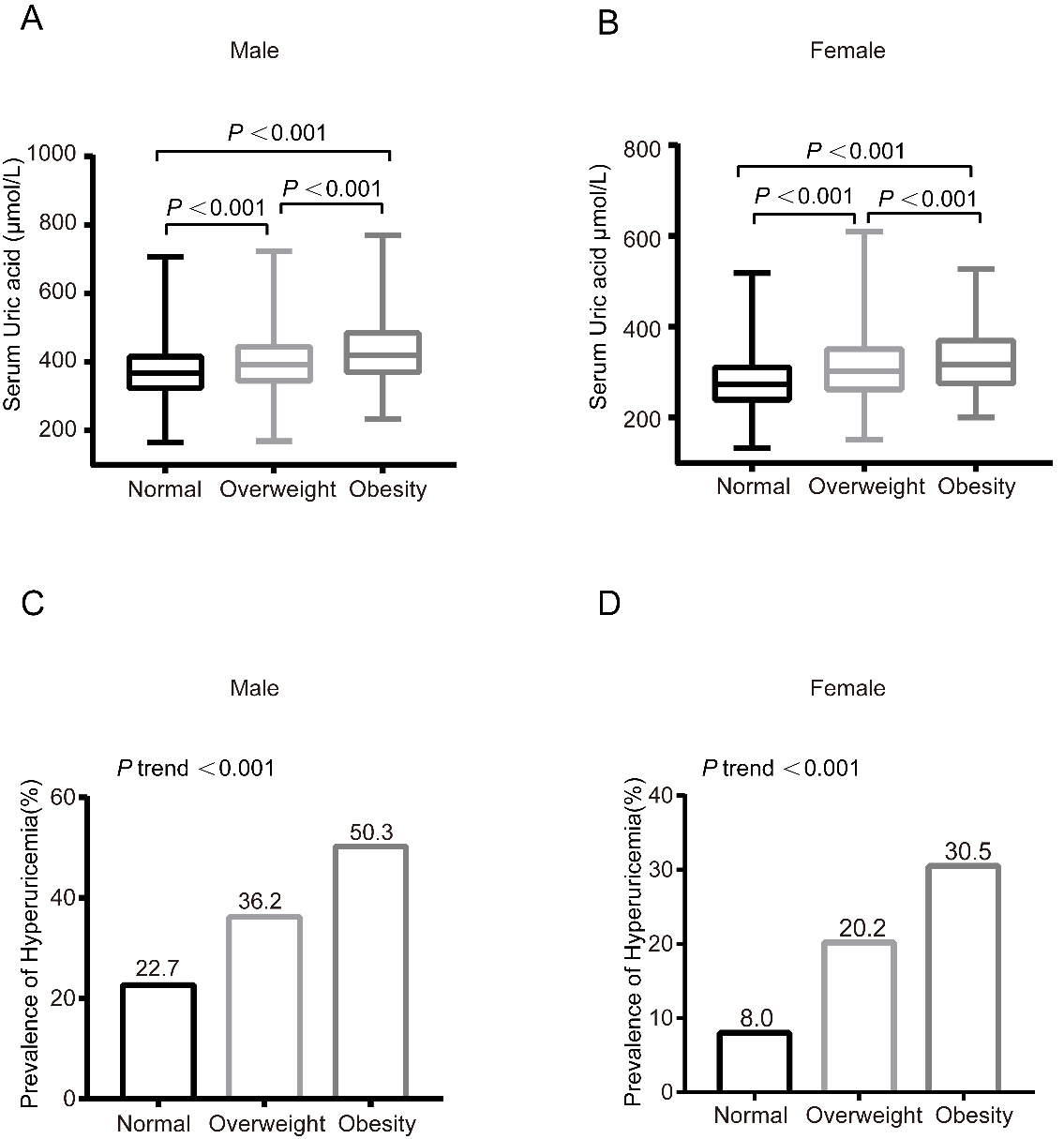
~~

**Supplementary Figure 3. The serum UA levels (A and B) and prevalence of hyperuricemia(C and D) across different BMI subgroups.** Data were expressed as median (upper and lower quartiles) or proportion (%). *P* trend: from test for linearity.


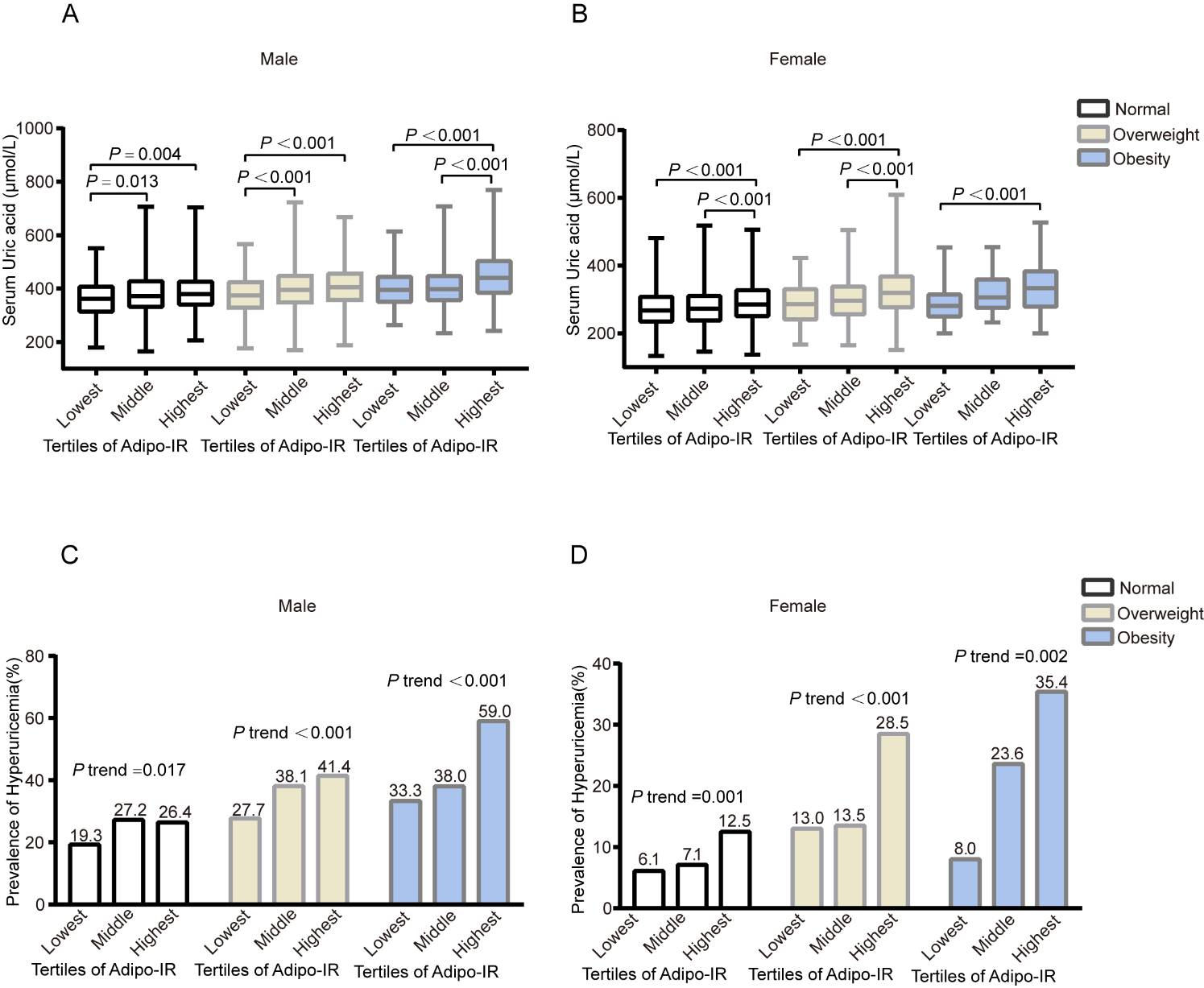


**Supplementary Figure 4. The serum UA levels (A and B) and prevalence of hyperuricemia(C and D) across the Adipo-IR tertiles in different BMI subgroups.** Data were expressed as median (upper and lower quartiles) or proportion (%).*P* trend: from test for linearity.

**
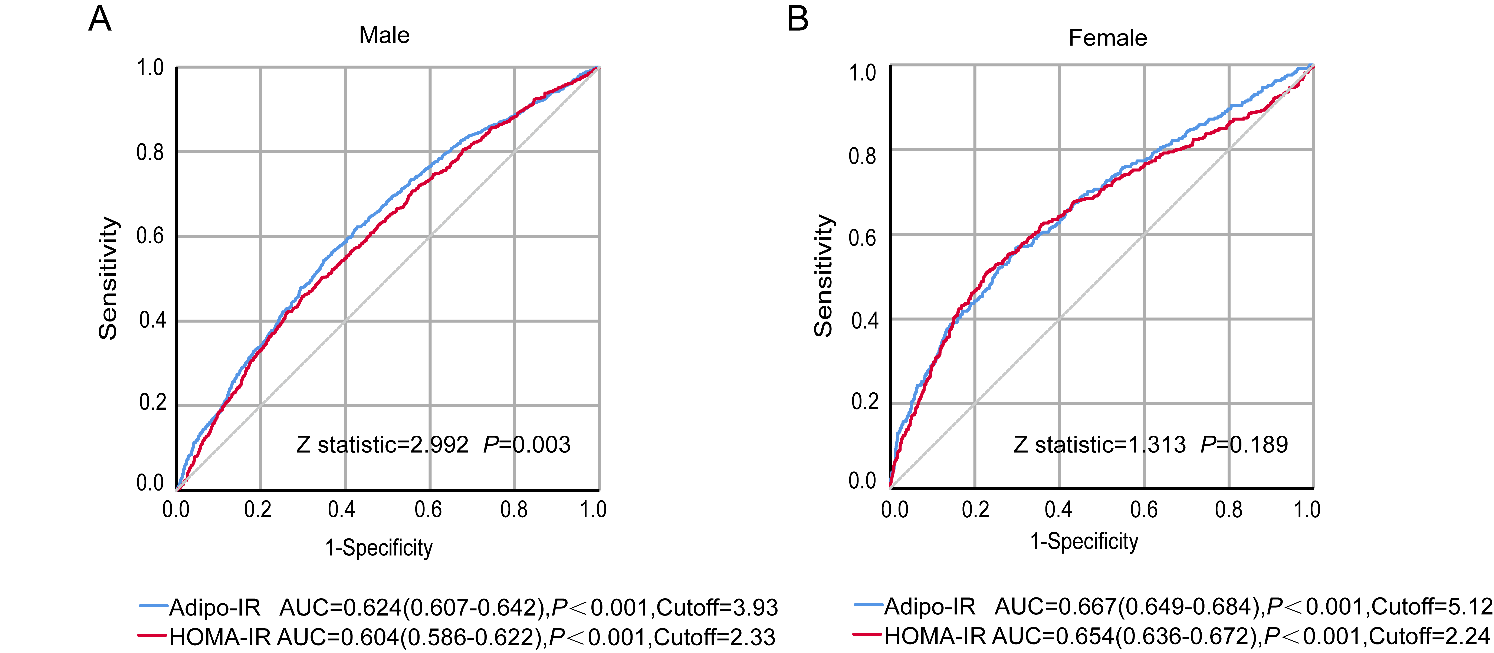
**

**Supplementary Figure 5. ROC curves of the Adipo-IR or HOMA-IR to hyperuricemia for males (A) and females (B).**
